# First episode of psychosis and transition to schizophrenia: the role of polypharmacy

**DOI:** 10.1192/j.eurpsy.2025.2269

**Published:** 2025-08-26

**Authors:** R. Softic, S. Osmanovic

**Affiliations:** 1 Psychiatry Clinic, University Clinical Center Tuzla, Tuzla; 2 Psychiatry, Health Center Brcko, Brcko, Bosnia and Herzegovina

## Abstract

**Introduction:**

The early stages after the onset of a first episode of psychosis (FEP) are crucial for the long-term outcome of the disease. Good outcome can be expected in <50% of patients, but three-quarters of all patients who experience a remission from a first episode of psychosis will have a recurrence of psychotic symptoms within a year of treatment discontinuation. Relapse prevention is key to preventing disease progression and further deterioration. Considerable number of patients experiencing a first episode of psychosis, will eventually transition to a diagnosis of Schizophrenia, so maintenance treatment should be the preferred option even in stable patients after a first episode of psychosis to remain in recovery. There is scarce information about differential effectiveness of antipsycotics in the long term. In such an atmosphere, the idea of polypharmacy with antipsychotics arises and may gain more supporters. Despite its obsolence, and unclear therapeutic benefits, as well as significant health risks, polypharmacy with antipsychotics is relatively common. This practice, due to unwanted effects, can lead to the arbitrary discontinuation of medication, and the consequent relapse of psychosis.

**Objectives:**

To determine the association of transition to schiyophrenia after the first episode of psychosis with a monotherapeutic or polypharmacy approach to the use of antipsychotics.

**Methods:**

A retrospective analysis of all hospitalized patients (87 patients, 65.5% were women), diagnosed with first episode of psychosis during a five-year period was conducted. The rate of relapse, and conversion to schizophrenia was analyzed in relation to the therapeutic approach (monotherapy vs polypharmacy with antipsychotics), within one year after the end of hospitalization due to the first episode of psychosis.

**Results:**

35.6% (31) of the subjects were treated with monotherapy. 25% (8) of them relapsed within a one-year period. 64.4% (56) of patients were treated with polypharmacy. 55.2% (48) of patients were treated with two antipsychotics, and 9.2% (8) with three. 75% (24) of subjects treated with polypharmacy had a relapse of psychosis within a year after discharge. There is a statistically significant difference between the groups of patients (p˂ 0.05).

**Conclusions:**

A significantly higher rate of relapse, and conversion to schizophrenia within a year after the end of hospitalization due to the first psychotic episode exists in subjects who were treated with two or more antipsychotics compared to subjects treated with monotherapy. The practice of polypharmacy with antipsychotics should remain reserved for individual, specially selected patients.

**Disclosure of Interest:**

None Declared

